# Expression of Interleukin-1ß and Interleukin-8 in Oral Potentially Malignant Disorders and Carcinomas

**DOI:** 10.3389/froh.2021.649406

**Published:** 2021-09-10

**Authors:** Jeaneth Lopez-Labady, Ronell Bologna-Molina, Mariana Villarroel-Dorrego

**Affiliations:** ^1^Dental School, Universidad Central de Venezuela, Caracas, Venezuela; ^2^Molecular Pathology Area, School of Dentistry, University of the Republic, Uruguay University of the Republic (UDELAR), Montevideo, Uruguay; ^3^Oral Histopathology Laboratory, Dental School, Universidad Central de Venezuela, Caracas, Venezuela

**Keywords:** IL-1β, IL-8, oral carcinoma, leukoplakia, oral lichen planus, inflammation

## Abstract

**Objective:** To evaluate interleukin-1ß (IL-1ß) and interleukin-8 (IL-8) epithelial expressions in potentially malignant disorders of the oral mucosa as malignant predictive markers.

**Study design:** About 55 tissues embedded in paraffin, comprising 15 oral lichen planus (OLP) lesions, 15 leukoplakias, 15 oral squamous cell carcinomas (OSCC), and 10 samples of normal oral mucosa were included in the study. IL-1ß and 8 expressions were assessed by immunohistochemistry using antibodies antihuman IL-1ß human (sc-7884, Santa Cruz® H-153) and antihuman IL-8 (ab7747, abcam®). The number of positive cells was compared using Student's *t*-test. Any *p*-value < 0.05 was considered statistically significant.

**Results:** Nuclear and cytoplasmatic keratinocyte staining were positive for both cytokines in all study groups. However, a statistically significant decrease was observed within all cases compared to normal mucosa, both staining for IL-1β and 8. Moreover, IL-8 showed significant differences between OLP and leukoplakia, and when compared to OSCC.

**Conclusions:** Oral epithelial expression of IL-1β and 8 seems to decrease when the malignant transformation of the oral mucosa increases.

## Highlights

- Normal oral keratinocytes can express IL-1β and 8.
- The nuclear expression of IL-1β and 8 are possible in oral keratinocytes.
- Expression of IL-1β and 8 are present in both leukoplakia and OLP, although variable.
- As OLP is an inflammatory disease, the expression of IL-1β and 8 are present, although variable. The absence of tissular IL-1β and 8 may lead to OSCC progression.
- A decrease of tissular IL-1β and 8 may lead to OSCC progression. The saliva scenario might be different and maybe not comparable.
- New studies are needed to correlate the observed findings with the prognosis in OSCC.

## Introduction

Currently, the term “precancerous” in oral mucosa has been replaced by potentially malignant disorders, recognizing the fact that not all disorders become oral carcinomas and malignant transformation may appear outside of the lesion area. Oral potentially malignant disorders include leukoplakia and proliferative verrucous leukoplakia, erythroplakia, submucous fibrosis, palatal lesions in reverse smokers, oral lichen planus (OLP), actinic cheilitis, lupus erythematosus, and most recently included chronic hyperplastic candidosis, oral lichenoid lesions, verrucous hyperplasia, and oral lesions of graft vs. host disease [1].

Leukoplakia and OLP are the most common potentially malignant disorders of the oral mucosa. Leukoplakia is defined by WHO as an oral white plaque of questionable risk having excluded known diseases or disorders that carry no increased risk for cancer [1]. OLP is a chronic mucocutaneous T-cell mediated inflammatory disease [1]. Although, it is defined by WHO as a potentially malignant disorder, there is some controversy, as a result of the malignant transformation, the rate ranges from 0% up to ~8% [1].

When any of those two disorders progress into cancer, it is done as an oral squamous cell carcinoma (OSCC), the most frequent head and neck malignancy. OSCC is the eighth most common type of cancer worldwide and the sixth most common cause of death of all malignancies, with a survival rate of around 40–50% in 5 years [2].

The role played by different cytokines in the transformation of leukoplakia and OLP into OSCC has been investigated mainly as salivary markers [3–8]. It has been shown that different tumors can produce cytokines in an autocrine manner and act by promoting angiogenesis and immunologic responses favoring the tumor [9–12].

Interleukin-1ß (IL-1β) and interleukin-8 (IL-8) are produced by a wide cell diversity, including oral keratinocytes [10, 12]. They have also been detected in high concentrations both in serum and saliva of patients with OSCC, suggesting they could serve as biomarkers for the disease [7, 8, 10].

Interleukin-1ß, a typical cancer-inflammation-linked cytokine, might play an important role in OSCC pathogenesis. Evidence shows IL-1β knockdown significantly inhibits OSCC cell growth and is regulated by genes, such as TGFβ [13]. Also, IL-1β has been identified as one of the key node genes in the tumor microenvironment during oral carcinogenesis [13].

Interleukin-8 is secreted by different stimulae among which IL-1 is included [14]. Higher levels of IL-8 have been found in the saliva of patients with OSCC, considering IL-8 a promising biomarker for oral malignant tranformation [14–18]. At this time, there is not enough evidence related to tissular inflammatory markers to be used for predictive purposes and therapeutic management of oral cancer. Therefore, the present study aimed to evaluate tissue expression of IL-1ß and 8 in leukoplakia, OLP, and OSCC.

## Materials and Methods

### Study Group

Biopsies of 55 subjects were included in this study. Bioethics approval for the study was obtained from the Bioethics Committee of Dental School, Universidad Central de Venezuela, under N° 0353–2012. Samples of OLP (*n* = 15), leukoplakias with histopathological evidence of mild to moderate epithelial dysplasia (*n* = 15), and well-differentiated OSCC (*n* = 15) were included after patient written consent. Normal gingival mucosa (*n* = 10) was obtained from healthy patients undergoing impacted third molar extraction, with no clinical signs of swelling or infection, and who gave informed written consent to participate in the study.

### Immunohistochemistry

Sections of 4 μm were cut for indirect biotin-streptavidin-peroxidase staining. For antigen retrieval, a pH 6.1 repair solution (DAKO®, Santa Clara, CA, USA) in a steamer was used for an hour. Tissue sections were incubated with rabbit primary polyclonal anti- IL-1ß human antibodies (Santa Cruz® H-153, sc-7884, Texas, TX, USA) at 1:50 dilution and anti-IL-8 human antibody (Abcam®, ab7747) at 1:25 dilution for 1 h. Detection and amplification system EnVision (DAKO®) was applied for 30 min. Slices were exposed to diaminobenzidine chromogen (DAKO®) and finally stained with hematoxylin. Pinolidal cyst and tonsillar tissue were used as positive controls for IL-1β and IL-8, respectively.

### IL-1ß and IL-8 Quantification

Slides were placed in an optic photomicroscope (OlympusCX41, San Diego, CA, USA) and observed under X10/0.25 magnifications. Digital images were taken from five different fields and saved as a jpeg file. A 6 × 6 grid was adapted to each image and examined for nuclear keratinocyte counting [19] using the digital image processing software ImageJ (1.46ª version). The mean of positive cells was calculated for each case.

### Statistical Analysis

Data obtained were analyzed statistically by using SPSS statistical software (version 18.0). The number of positive cells was expressed by mean ± SD and compared using Student's *t-*test for independent samples. *P-*values <0.05 were considered statistically significant.

## Results

**Figure 1:**
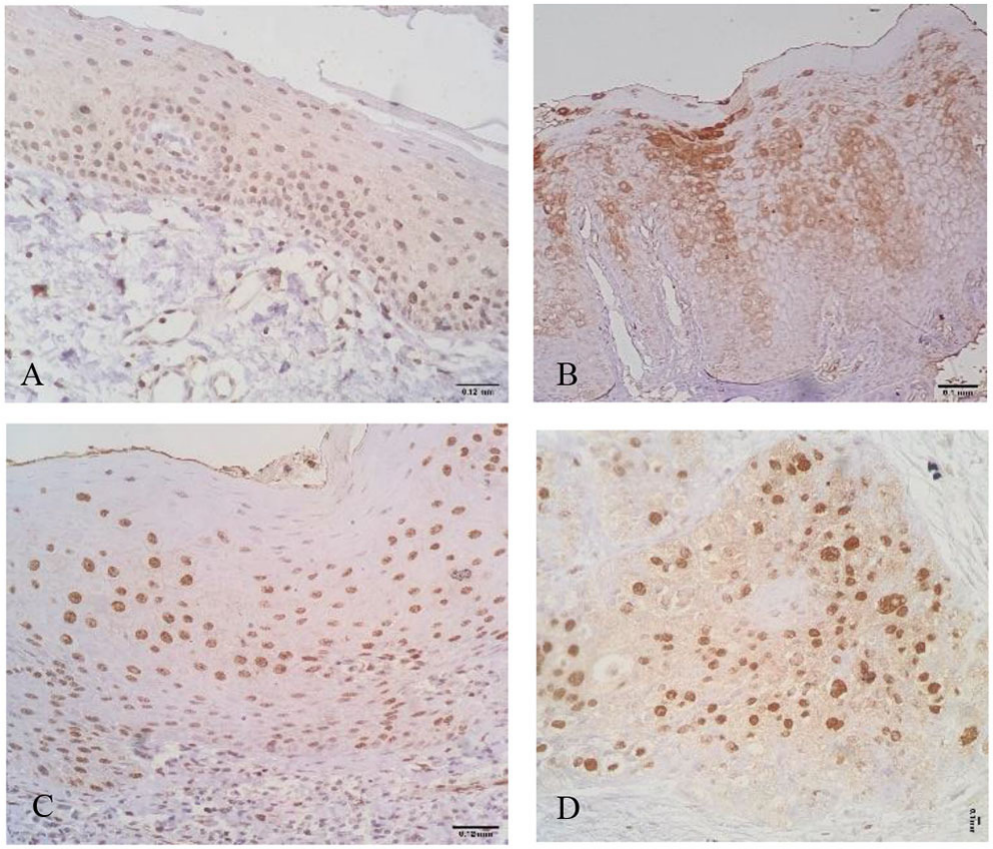
Interleukin-1β (IL-1β) + cells in oral healthy mucosa **(A)**, leukoplakia **(B)**, oral lichen planus (OLP) **(C)**, and oral squamous cell carcinoma (OSCC) **(D)**. Note different patterns of expression where cytoplasmatic and nuclear expressions are evident.

**Figure 2:**
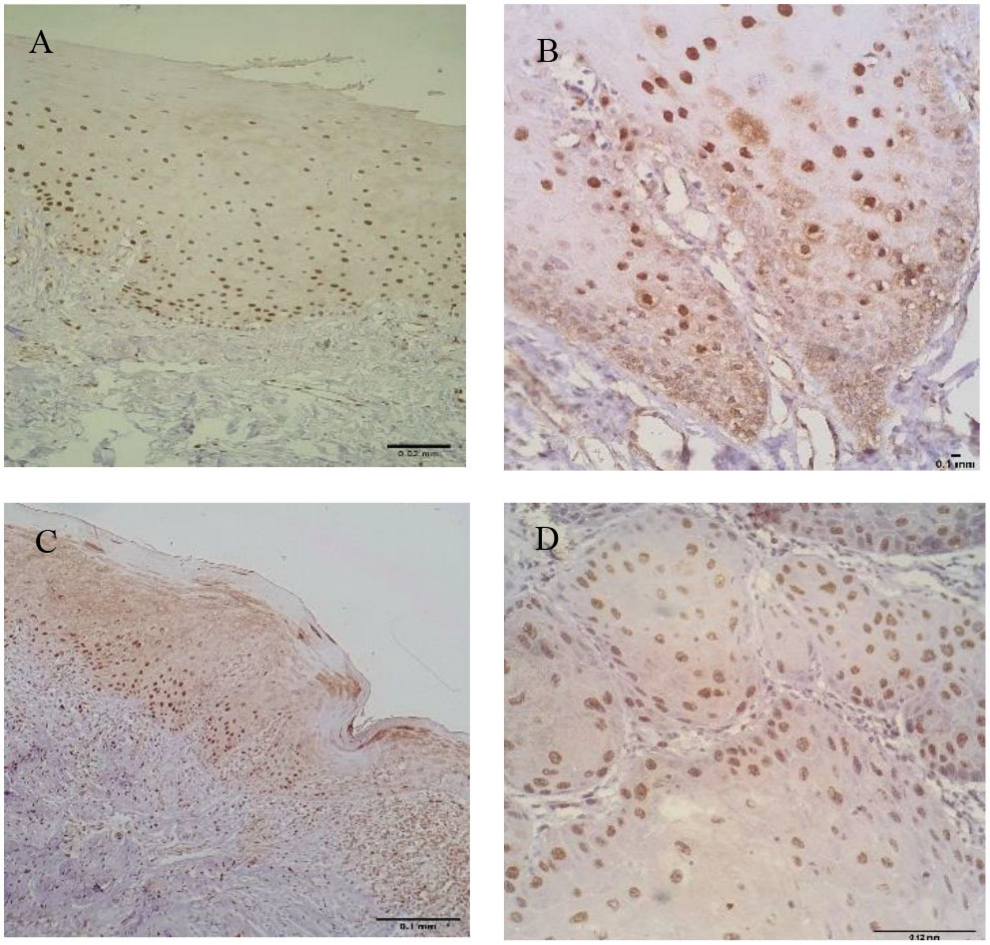
Keratinocytes expressing interleukin-8 (IL-8) in oral healthy mucosa **(A)**, leukoplakia **(B)**, OLP **(C)**, and OSCC **(D)**. Nuclear and cytoplasmatic staining was observed in all groups.

Immunostaining for IL-1β and IL-8 was observed in the nucleus and cytoplasm of keratinocytes (Figures 1, 2).

Interestingly, the highest expression of IL-1β was observed in oral healthy mucosa (mean of 329.25 ± 90.55 cells/field). Lower IL-1β positive cells were observed in leukoplakias (224.60 ± 161.05 cells/field), OLP (111 ± 101.752 cells/field), and OSCC (132.07 ± 121.951 cells/field) (Table 1, Figure 1).

**Table 1:** Interleukin-1β (IL-1β) and Interleukin-8 (IL-8) keratinocyte expression.

**Interleukin**	**Mean ± SD positive cells/ field**				***p*** **-value**
	**Oral normal mucosa**	**OLP**	**Oral leukoplakia**	**OSCC**	
IL-1β	329.25 ± 90.55	111 ± 101.75	224.60 ± 161.05	132.07 ± 21.95	0.06
IL-8	389.8 ± 84.5	230.02 ± 227.67	413.87 ± 250.78	255 ± 193.9	0.046

When individual groups were compared, statistically significant decreased IL-1β expression was observed between healthy patients and OLP (*p* = 0.0001) and OSCC (*p* = 0.001) groups (Table 2).

**Table 2:** Interleukin-1β *p*-values comparison among groups.

**Oral lesion**	**Oral normal mucosa**	**OLP**	**Oral leukoplakia**	**OSCC**
Oral normal mucosa	-	0.0001^*^		0.001^*^
OLP	0.0001^*^	-	0.029^*^	0.611
Oral leukoplakia	0.106	0.029^*^	-	0.087
OSCC	0.001^*^	0.611	0.087	-

* *Statistically significant*.

In relation to IL-8, the highest mean of positive cells was observed in leukoplakias (413.87 ± 250.789 cells/field) followed by normal oral mucosa (389.80 ± 84.507 cells/field), OSCC (255 ± 193.90 cells/field), and finally OLP (230.02 ± 227.67 cells/field) (*p* = 0.046) (Table 1, Figure 2).

Interleukin-8 was statistically decreased in OSCC when compared to the normal oral mucosa (*p* = 0.026), and conversely statically increased in leukoplakias (*p* = 0.009) (Table 3).

**Table 3:** Interleukin-8 *p*-values comparison among groups.

**Oral lesion**	**Oral normal mucosa**	**OLP**	**Oral leukoplakia**	**OSCC**
Oral normal mucosa	-	0.101	0.009^*^	0.026^*^
OLP	0.101	-	0.342	0.958
Oral leukoplakia	0.009^*^	0.342	-	0.043^*^
OSCC	0.026^*^	0.958	0.043^*^	-

* *Statistically significant*.

## Discussion

Interleukin-1β is a pro-inflammatory cytokine that is implied the most in oral carcinogenesis [8, 20]. The research evidenced interesting findings related to the immunohistochemical analysis of this cytokine; nuclear and cytoplasmic keratinocyte staining was observed in all the study groups, and IL-1β was increasingly expressed by oral healthy mucosa.

Nuclear keratinocyte expression of IL-1 and IL-8 has been observed before [21], moreover, IL-1α and IL-1β have been observed *in vivo* and *in vitro* in gingival keratinocytes, probably as a result of permanent bacterial response [22–24]. It is known that inflammasome activation induces caspase-1 dependent secretion of the pro-inflammatory cytokine IL-1β in keratinocytes. Gingival keratinocytes also can produce IL-8 in response to IL-1β maintaining tissue micro-environment [22–24]. The normal mucosa samples were originated by periodontal tissue and that could explain the persistent expression of both cytokines under “healthy” or insignificant clinical inflammatory circumstances.

The oral environment is formed by a series of elements that make it an immunological organ, such as the presence of lymphoid tissue associated with the mucosa, microbiota, saliva with components, such as secretory IgA and a large variety of biomarkers [18]. Additionally, it has been shown that oral keratinocytes can express molecules of histocompatibility type II complex, which make it behave as an antigen-presenting cell [25], and make it capable of expressing and secreting pro-inflammatory cytokines such as IL-1β and IL-8 [26, 27]. The use of gingival mucosa, as mentioned, could imply a pro-inflammatory environment, which may not represent a strictly healthy control, although, the results favor a decreased expression than in oral potentially malignant disorders studied.

Wu et al. [13] observed, in a rat model, keratinocyte IL-1β positivity in healthy oral mucosa, leukoplakia, and OSCC; however, staining was more significant in cases of the disease, when comparing with healthy tissue, suggesting that cytokines increase is more closely related to malignant transformation.

Taking into account that the IL-1β expression and secretion by oral keratinocyte is inflammasome-dependent, IL-1 may have a role in cell death activation through pyroptosis, the process currently recognized as necessary for cell exchange and protection of oral mucosa epithelium [28]. Mechanism of cell death derived from the activation of the inflammasome, which involves IL-1β secretion as an oncosupressive effect [28], might explain the observations in this study, where a significant reduction in IL-1β expression by keratinocyte was observed. This might suggest that loss of IL-1β implies pyroptosis reduction, favoring cell proliferation, observed in OLP and leukoplakia, which is a determining fact of OSCC pathogenesis.

A dual role for IL-1β in carcinogenesis, tumor growth, invasion, and metastasis has also been suggested [28, 29]. Gasparoto et al. [30] chemically induced squamous cell carcinomas in mouse skin and investigated the effect of the inflammasome on the appearance and tumor development. The authors observed that proteins derived from the inflammasome were involved in the protection against squamous cell carcinoma since their loss hinders the development of the antitumor response. On the other hand, and consistent with the dual role of this cytokine, Dantas et al. [31] observed how IL-1β immunostaining was significantly higher in metastases of oral carcinomas and lymph nodes than in squamous epithelium of tumor resection margins; while Singh et al. [32] described how the immunostaining for IL-1β in non-metastatic oral carcinomas was weak in more than half of the cases. Therefore, the presence of IL-1β in oral epithelial tissues remains controversial.

Interleukin-8 is also related to carcinogenesis. It has an important angiogenic and chemotactic performance, being produced by a wide group of cells, the keratinocytes among them. Additionally, the expression of nuclear and cytoplasmatic IL-8 in OSCC keratinocytes has been described [33].

Studies assessing IL-8 in potentially malignant disorders and OSCC have been reported in saliva and serum [16, 17], however, in oral mucosa by immunohistochemical staining are scarce and results seem different. Jenkins et al. [34] assessed the intensity of the NF-κB primary transcription factor and IL-8 expression in samples of esophageal adenocarcinoma, Barrett esophagitis (as premalignant precursor lesion), and squamous tissue next to these lesions and observed IL-8 diminish, similar to results obtained in the study.

Immunohistochemical behavior of IL-8 was similar to the one observed with IL-1β, in this sense, previous studies [11, 18, 24, 25] have shown that the IL-1ß induction is within the costimulating factors which produce IL-8, so these results may have a direct relationship with this fact. Taking into account that IL-8 is a chemoattractant cytokine, for cells such as polymorphonuclear neutrophils and lymphocytes [11, 35], its absence or reduction in the expression of disease cases, mainly OSCC, could represent a loss of antitumor immune vigilance favoring carcinogenic activity.

Lee et al. [35] studied the effect of IL-8 in the tumorigenesis of human ovarian cancer in mice. Cells transfected with IL-8 exhibited poor growth. Authors related the reduction of growth and cell proliferation with the leakage of activated neutrophils, suggesting the release of oxygen and proteases might produce the death of tumor cells.

Decrease expression observed with IL-8 can be related to the IL-1ß which induces its synthesis. This finding might imply a loss of chemotactic activity reducing antitumor function induced by polymorphonuclear neutrophils and lymphocytes. IL-1ß immunohistochemical findings allow suggesting that patients with OSCC might exhibit epithelial loss of this cytokine, which might be related to deactivation of the inflammasome at the keratinocyte producing not only the reduction of its expression and secretion but also the reduction of pyroptosis favoring the carcinogenesis process.

In OLP, some studies have described abnormal expression patterns of several inflammation-related cytokines (including IL-1 β and IL-8) in tissues, saliva, and serum from patients [7, 12, 36]. In this study, OLP showed the lowest cytokine expression probably related to the chronic inflammatory nature of the pathology.

While IL-1β and IL-8 were decreased in OLP lesions, various studies have consistently shown increased levels of IL-8 in serum and saliva from patients with OLP [7, 12, 36, 37]. This disparity between tissues and fluids indicates that ILs may function mainly in the systemic immune response of patients with OLP rather than in the local lesions.

Additionally, it has been described that the level of salivary IL-8 is lower in OLP with epithelial dysplasia than patients with OSCC [12, 37], indicating that salivary IL-8 level might be useful, non-invasive method for monitoring the malignant transformation of OLP.

## Conclusions

Oral epithelial expression of IL-1β and 8 seem to decrease when the malignant transformation of the oral mucosa increases. IL-1β may play a role in oral carcinogenesis *via* keratinocytes.

A significant discovery of the results was observed in gingival keratinocytes, showing expression of both cytokines under normal, or mild inflammatory conditions.

## Statement of Clinical Relevance

Loss of IL-1β and 8 in potentially malignant disorders, as leukoplakia and OLP, might suggest the start of a malignant transformation process.

## Data Availability Statement

The raw data supporting the conclusions of this article will be made available by the authors, without undue reservation.

## Author Contributions

All authors contributed to the design and implementation of the research, to the analysis of the results, and to the writing of the manuscript.

## Conflict of Interest

The authors declare that the research was conducted in the absence of any commercial or financial relationships that could be construed as a potential conflict of interest.

## Publisher's Note

All claims expressed in this article are solely those of the authors and do not necessarily represent those of their affiliated organizations, or those of the publisher, the editors and the reviewers. Any product that may be evaluated in this article, or claim that may be made by its manufacturer, is not guaranteed or endorsed by the publisher.
